# Application of Soft-Clustering to Assess Consciousness in a CLIS Patient

**DOI:** 10.3390/brainsci13010065

**Published:** 2022-12-29

**Authors:** Sophie Adama, Martin Bogdan

**Affiliations:** Department of Neuromorphic Information Processing, Leipzig University, Augustusplatz 10, 04109 Leipzig, Germany

**Keywords:** completely locked-in syndrome, complexity, connectivity, consciousness, electrocorticogram, features extraction, soft-clustering, spectral analysis

## Abstract

Completely locked-in (CLIS) patients are characterized by sufficiently intact cognitive functions, but a complete paralysis that prevents them to interact with their surroundings. On one hand, studies have shown that the ability to communicate plays an important part in these patients’ quality of life and prognosis. On the other hand, brain-computer interfaces (BCIs) provide a means for them to communicate using their brain signals. However, one major problem for such patients is the difficulty to determine if they are conscious or not at a specific time. This work aims to combine different sets of features consisting of spectral, complexity and connectivity measures, to increase the probability of correctly estimating CLIS patients’ consciousness levels. The proposed approach was tested on data from one CLIS patient, which is particular in the sense that the experimenter was able to point out one time frame Δt during which he was undoubtedly conscious. Results showed that the method presented in this paper was able to detect increases and decreases of the patient’s consciousness levels. More specifically, increases were observed during this Δt, corroborating the assertion of the experimenter reporting that the patient was definitely conscious then. Assessing the patients’ consciousness is intended as a step prior attempting to communicate with them, in order to maximize the efficiency of BCI-based communication systems.

## 1. Introduction

Patients with locked-in syndrome (LIS) are incapable of producing speech and voluntary limb movements. They are however perfectly conscious and their cognitive functions are unaltered [1,2]. Communication are usually carried out using eye movements [3]. This means of communication becomes impossible once the patient enter a completely locked-in state (CLIS) in which no residual muscle movement prevail [4]. At this point, there is no external way of determining if they are conscious or not at a specific time. The most common cause of LIS is traumatic brain injury [5]. However, it can also be caused by a neurological disease, in particular Amyotrophic Lateral Sclerosis (ALS) [6]. Due to the lack of external manifestation of conscious states, patients in such condition are often misdiagnosed as suffering from disorders of consciousness (DoC) [7].

DoC states encompass those in which an individual’s consciousness is impaired. It usually occur after a brain injury, and one can distinguish coma, vegetative state (VS) or Unresponsive Wakefulness Syndrome (UWS), and Minimally Conscious State (MCS) [3]. Patients can be in coma for two to four weeks during which they are “unarousable”. This state is characterised by an absence of spontaneous eyes opening and muscle movements [2,8]. If and when patients emerge from this state, they can enter either a locked-in or a vegetative state, which in turn can transition to a MCS, or in the worst case scenario, into permanent VS and/or death. Although not a DoC, CLIS appear to be similar to VS. This turn of event can then lead to misdiagnosis [9], which denies the patients the appropriate medical care, and reduces the chances of rehabilitation as well as their quality of life [10]. Moreover, every chance to communicate with the patients is lost, further diminishing their quality of life.

There are only a handful of researches dealing with the assessment of CLIS patients’ consciousness. This is certainly because of the rarity of this disease, which according to OrphaNet (OrphaNet is a database for rare disease and orphan drugs. https://www.orpha.net/ (accessed on 22 November 2022), has a prevalence of less than 1/1,000,000. Furthermore, these studies do not detect the CLIS patients’ consciousness per se, rather they aim at differentiating them from DoC patients. To that end, they rely on the patients’ active participation by the means of event-related potentials, which easily fatigue them [11]. This is done for instance using P300 elicited by the subject’s own name (SON) [12,13]. Most of the time, P300 responses were observed for all LIS patients, as opposed to the patients with DoC. When combined with Steady-State Visual Evoked Potentials (SSVEPs) in a visual hybrid Brain-Computer Interface (BCI), the results of one LIS patient was comparable to that of a healthy subject [14]. Furthermore, most researches do not evaluate patients’ readiness or willingness to perform the tasks. One exception is Guger et al. that designed a task to evaluate the states of 6 chronic LIS patients using a vibro-tactile oddball paradigm to elicit P300, before attempting a binary (*yes* and *no*) communication with them [15]. 5 of the patients were able to elicit P300 responses with an accuracy of 100% for 4 of them.

Although these results suggest that by successfully performing the tasks presented to them, one can assume that these patients were conscious. However, they are unable to overtly communicate, and more importantly, there are no ground truth related to the state they might be in.

This work reports the case of a CLIS patient, which consciousness level was determined by extracting several features from his (mostly) resting state Electrocorticogram (ECoG), and using them as input to two clustering analysis approaches that afterwards issue an estimation of his consciousness level. By doing so, the prospect is to maximise the probability of correctly determining the patient’s actual states at a specific time. Indeed, a signature of probable consciousness detected by one feature may be missed by another.

This paper is organised as follows: in Section 2, the patient as well as the data recorded from him is first introduced. Then the process of determining his consciousness level is briefly described. Subsequently, the results are presented in Section 3 and discussed in Section 4.

## 2. Materials and Methods

### 2.1. Patient Description

**Figure 1:**
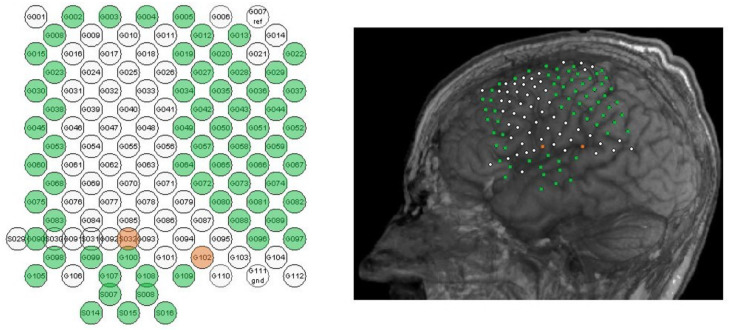
ECoG channels positions. **Left**: channel names, with functional recording channels shown in green, and ground (S032) and reference (G102) channels in orange. **Right**: spatial location of surgically implanted ECoG grid electrodes.

The data was recorded from a 40-year-old male CLIS patient. It consists of ECoG, also known as intraoperative cortical electroencephalogram, which is an invasive way to record electrical brain activity. This patient was first diagnosed with amyotrophic lateral sclerosis (ALS) in 1997 and entered CLIS eleven years later [16]. The dataset comprises 24 consecutive one-hour recordings (i.e., 24 h in total) and was acquired with a 64-channel amplifier (BrainAmp from Brainproducts GmbH, Munich, Germany) at a sampling rate of 500 Hz. The ECoG grid electrodes were surgically placed on the patient’s left frontal and parietal lobes [17], as illustrated in Figure 1, which also shows the specific channels names and locations.

An auditory paradigm was performed, in which the patient was asked questions requiring a *yes* or *no* answer. These questions covered a range of topics such as his mood, feelings and his physiological status. The questions were also paired, which means that for each question requiring a positive answer, there is a corresponding one that requires a negative answer. The pairs of questions can be for instance: “You feel good today?”/“You feel bad today?” or “Are you German?”/“Are you Dutch?”. The answers to all questions are known by the family and/or caregivers. This enables the experimenters to use them to train a classifier and use it later to predict the patient’s answers to open questions. The entire course of the interaction between the patient and the experimenter during the experiment can be downloaded at: Supplementary Materials

### 2.2. Methods Description

**Figure 2:**
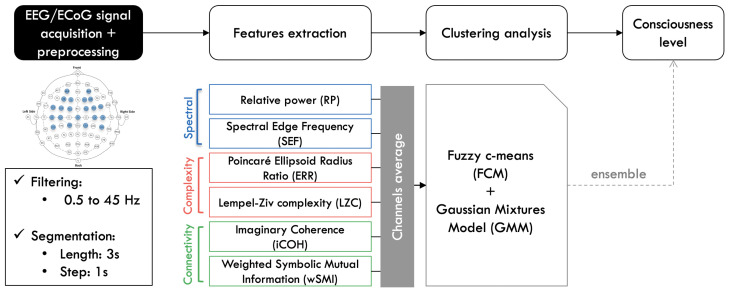
Signal processing and analysis pipeline. The ECoG signal is filtered and segmented, before different features were extracted from it. Each feature is then averaged across all channels before performing the clustering analysis. The consciousness level of the patient is then inferred from the obtained results [20].

All analysis were performed using MATLAB, the FieldTrip toolbox [18], and also custom written scripts. The modus operandi of the method illustrated in Figure 2 was first introduced in [19], afterwards implemented and described in more details in [20].

Given the state of the patient, no artefacts removal was performed on the data. At first, the ECoG was filtered from 0.5 to 45 Hz using a third order Butterworth filter. All features were computed for all channels in segments of 3-s length, with a sliding window of 1-s. The obtained outcomes were subsequently averaged across all of them, and clustered using two different soft clustering approaches.

#### 2.2.1. Features Computation

After filtering and segmenting the ECoG data, diverse types of features were computed from it in order to maximise the probability of correctly determining the CLIS patient state.

##### Spectral Features

Spectral features of a signal encompass its frequency and power characteristics. In case of brain signals, information about the brain states can be obtained from the values of the different frequency powers [21]. Two spectral measures were used in this research, namely the Relative Power (RP) and the Spectral Edge Frequency (SEF).

RP of θ (0 to 4 Hz) and β (12 to 30 Hz) were used since they proved to be very efficient in distinguishing the levels of consciousness between Minimally Conscious State (MCS) and Unresponsive Wakefulness Syndrome (UWS) patients with disorders of consciousness [22]. An increase of θ power is observed during verbal and spatial memory tasks [23]. Moreover, the recovery of consciousness after anaesthesia is revealed by a global increase of the θ power and also the γ power and coherence [24]. Additionally, when the brain is engaged in information processing, the cortical neurons are highly activated and relatively asynchronous called β rhythms [21,25]. Consequently, a conscious state is hypothesized to be expressed by an increase of both the θ and β powers.

For a signal x(t), RP is computed as:

(1) $$RP=\frac{\sum_{f=f_{1}}^{f_{2}}S_{x}\left(f\right)}{\sum_{f=f_{l}}^{f_{h}}S_{x}\left(f\right)}$$

where: $f_{1}$ and $f_{2}$ specify respectively the lower and upper limits of the frequency band of interest. $f_{l}=0$ Hz and $f_{h}=45$ Hz (upper limit of the cut-off frequency during filtering) in this particular case, and $S_{x}\left(f\right)$ is the power spectral density (PSD) of the signal x(t) at the frequency *f* [26]. The PSD was estimated using the MATLAB function pwelch with a Hamming window of 1/8 size of the data segment and a 50% overlap, using the Welch method [27].

SEF is the a threshold value of the frequency under which a specific fraction *r* of the signal power is contained [28,29]. It is calculated using Equation (2), with r=95% (SEF95). A lower SEF95 value characterises a deeper level of anaesthesia [30]. Plus, light anaesthesia is indicated by SEF95 higher than 15 Hz, i.e., in the β band, while frequencies lower than 7 Hz imply deep anaesthesia [31]. Thus, higher values of the SEF usually characterise a conscious state.

(2) $$\sum_{f=0}^{SEF_{r}}S_{x}\left(f\right)=r\sum_{f=0}^{Fs/2}S_{x}\left(f\right)$$

where: $F_{s}$ is the sampling frequency, and $S_{x}\left(f\right)$ is the PSD of the signal x(t) at the frequency *f*. The obtained value was furthermore normalised by dividing it to the upper limit of the critical frequency (45 Hz) during filtering.

##### Complexity Features

A complexity measure is a quantity that assess how sophisticated the structure of a biological system is. Signals with a certain uniformity have low complexity, while irregular signals have larger values. Besides, an activated brain produces largely complex signals [32]. Hence, conscious states are supposed to have higher complexity. Two measures of complexity were used: the Ellipsoid Radius Ratio (ERR) of the Poincaré plots and the Lempel-Ziv Complexity (LZC).

To build the Poincaré plot of a signal $X=x_{1},\ldots ,x_{N}$, each data point $x_{k}$ is plotted again its delayed version $x_{k+\tau }$. The value of the delay τ is chosen so that it is 1/5 to 1/4 of the dominant cycle period [33]. ERR is the ratio SD1/SD2 where SD2 and SD1 are respectively the standard deviation of the points along the line of identity, and perpendicular to the line of identity (cf. Equation (3)) [34].

(3) $$ERR=\frac{SD1}{SD2}=\frac{\frac{\sqrt{2}}{2}SD\left(x_{k}-x_{k+\tau }\right)}{\sqrt{2SD{\left(x_{k}\right)}^{2}-\frac{1}{2}{\left(x_{k}-x_{k+\tau }\right)}^{2}}}$$

On one hand, random signals are represented by a round oval pattern, which would correspond to ERR≈1. On the other hand, signals with linear features are illustrated by a more elongated shape [33]. The values of SD1 and SD2 were computed using the extended Poincaré plot algorithm developed in [35].

LZC is a complexity measure that was developed by Abraham Lempel and Jacob Ziv [36] to evaluate repetitiveness in binary sequences. Here, the data should be transformed into a binary sequence before the LZC could be computed. The algorithm then counts the number of distinct patterns in the data. High degree of randomness is characterised by a large number of different sub-sequences in the binary sequence, hence the greater the value of the Lempel-Ziv complexity [37,38].

##### Connectivity Features

Brain connectivity measures the interaction between two brain regions or between signals recorded from two channels. The connectivity features used in this research consist of the imaginary part of the coherency (iCOH), which assess the linear relationship between two entities, and the weighted Symbolic Mutual Information (wSMI) that, in addition to linear connections, also determines the non-linear relations. The θ band play an important part in working memory [23]. Additionally, the coherence in the lower frequency bands decrease during periods of unresponsiveness in healthy subjects under anaesthesia [39]. On the other hand, wSMI also assesses more accurately and robustly the long-range connectivity patterns theoretically related to consciousness [22]. And higher values of the wSMI in the θ band characterise higher levels of consciousness [40]. Accordingly, only connectivity in the θ band was employed.

Coherency can be used to determine the relative timing of activity between two brain regions and also their phase consistency [41,42,43,44]. Its value is complex, but using only the imaginary part allows to avoid volume conduction problems [43].

(4) $$iCOH=ℑ\left(C_{xy}\left(f\right)\right)=ℑ\left(\frac{S_{xy}\left(f\right)}{\sqrt{S_{xx}\left(f\right)\cdot S_{yy}\left(f\right)}}\right)$$

where $S_{xy}\left(f\right)$ is the cross power spectral density of the signals, and $S_{xx}\left(f\right)$ and $S_{yy}\left(f\right)$ are the auto power spectral density of *x* and *y* respectively [45].

Functional connectivity is the temporal coherence between the activities of different brain areas [46]. Higher values of the coherency characterise an increased functional interaction between the underlying neuronal networks [47]. Only the degree of relationship between the different pairs of channels is of interest, so the absolute value |iCOH| was used. For each data segment, a connectivity matrix representing the coupling between all pairs of channels is then obtained.

On the other hand, wSMI evaluates the degree to which two signals present nonrandom joint fluctuations which suggest information sharing. To compute its value between two time series *x* and *y*, they are first converted into sequences of discrete symbols $\left(\hat{x},\hat{y}\right)$ which values depend on a specific number *k* of successive time points distant by a temporal separation of elements τ [48]. Practically, k=3, leading to a total of 3!=6 different potential symbols [48]. The value of τ is chosen depending on the frequency band of interest: smaller values emphasize higher frequencies [49]. wSMI was estimated using Equation (5):

(5) $$wSMI\left(x,y\right)=\frac{1}{\log(k!)}\sum_{\hat{x}\in \hat{X}}^{}\sum_{\hat{y}\in \hat{Y}}^{}w\left(\hat{x},\hat{y}\right)p\left(\hat{x},\hat{y}\right)\log\left(\frac{p(\hat{x},\hat{y})}{p\left(\hat{x}\right)p\left(\hat{y}\right)}\right)$$

where $p(\hat{x},\hat{y})$ is the joint probability of co-occurrence of symbol $\hat{x}$ and symbol $\hat{y}$, $p(\hat{x})$ and $p(\hat{y})$ are the probabilities of those symbols in each respective signal. To reduce the computational time, the ECoG data was down-sampled from 500 Hz to 200 Hz. Afterwards, wSMI was computed using custom written MATLAB scripts. Similarly to iCOH, a connectivity matrix is obtained for each data segment.

#### 2.2.2. Data Clustering and Consciousness Level Assessment

The features were computed for all channels or pairs of channels and averaged across them. For the connectivity measures in particular, this average is obtained by calculating the mean of the lower part of their respective connectivity matrices without the diagonal. After averaging and before clustering analysis, the feature vector was normalised between 0 and 1. Two soft clustering analyses were applied to the extracted features: Fuzzy c-means (FCM) and Gaussian Mixture Models (GMM), which outputs were combined via an average ensemble [50,51] to obtain a unique value estimating the patients’ consciousness levels. Specifically, if $P(c,m_{1})$ represents the probability that the object *i* is a member of the cluster *c* in partition $m_{1}$, and $P(c,m_{2})$ the probability that the same object belongs to the cluster *c* in partition $m_{2}$, the average ensemble is obtained using Equation (6):

(6) $$P_{avg}\left(c,m_{1}m_{2}\right)=avg\left(P\left(c,m_{1}\right),P\left(c,m_{2}\right)\right)$$

Soft-clustering attributes a membership degree (from 0 to 1) to each cluster to each data point. The sum of the membership degrees to all clusters equals 1 [52]. FCM is a soft version of the *k*-means algorithm. It operates by introducing a fuzziness factor to obtain the membership degrees [52,53,54].

GMM on the other hand uses a Gaussian mixture distribution, that the clustering method attempts to recover, to model the data. The parameters of the model is estimated using an Expectation-Maximisation (EM) algorithm [55]. The goal in this research is to separate the features into two clusters corresponding to consciousness and unconsciousness respectively. The consciousness level is determined as the value of the degree of membership of each data point to the cluster corresponding to a conscious state. The characteristics of this cluster are determined according to the assumption that higher values of the selected features are representative of higher levels of consciousness.

On one hand, to implement the FCM clustering approach, the MATLAB function fcm was applied to the data with the specified parameters: N=2 clusters, the fuzzifier parameter *m* was set to 2 as recommended by previous research [52], the maximum number of iterations was fixed at 1000 and the minimum improvement in objective function between two consecutive iterations ϵ at $10^{-5}$. The algorithm then returns N=2 clusters centres for each dimension of the feature vector. On the other hand, the MATLAB function fitgmdist was used to fit GMMs to the data using the EM algorithm and the same parameters as with the FCM clustering analysis. In addition, MATLAB posterior function of the Statistics and Machine Learning Toolbox was used to estimate the component-membership posterior probabilities [56].

## 3. Results

The ECoG data was recorded during 24 h, from 00:34 to 00:34${}^{+1}$. The previously described experiment was carried out from 14:50 to 17:00 (delimited by red plain vertical lines). In the following figures, the red shaded area between 15:34 and 16:14 delimit the time frame when the experimenter asserted that patient GR was correctly answering the questions asked to him. Noisy channels were removed from the analysis. This concerns channels G008, G012, G028, G034, and G080 (cf. Figure 1) with signal amplitudes larger than 200 mV. The features were then computed on the remaining 59 channels and subsequently averaged across all of them.

**Figure 3:**
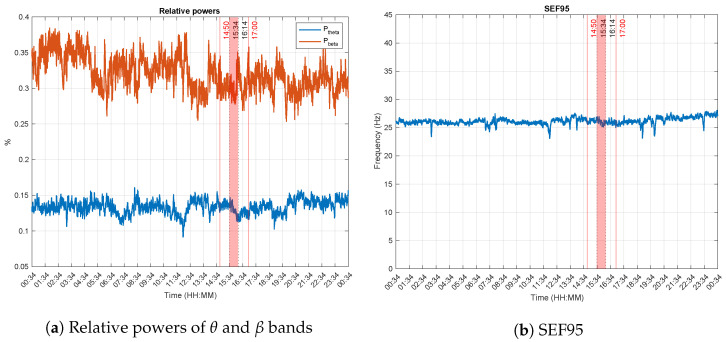
Spectral features. The experiment was performed between 14:50 and 17:00 (red vertical lines). The dotted lines at 15:34 and 16:14 delimit Δt, the time during which the experimenter reported that the patient was correctly answering the questions he was asked. Increases in the θ and β powers indicate an increased consciousness level and inversely. The average value of the SEF95 is above 25 Hz (β band), suggesting that the patient was conscious during the whole recording.

Figure 3 illustrates the spectral features. More particularly, Figure 3a displays the variations of the relative powers of θ and β bands. A decrease (resp. increase) can be observed in the course of the experiment delimited by the red vertical lines. SEF95 values are relatively steady during the whole recording as can be seen in Figure 3b), with an average value of 26 Hz throughout it all. This value is well above the β band, suggesting that the patient was conscious all along.

**Figure 4:**
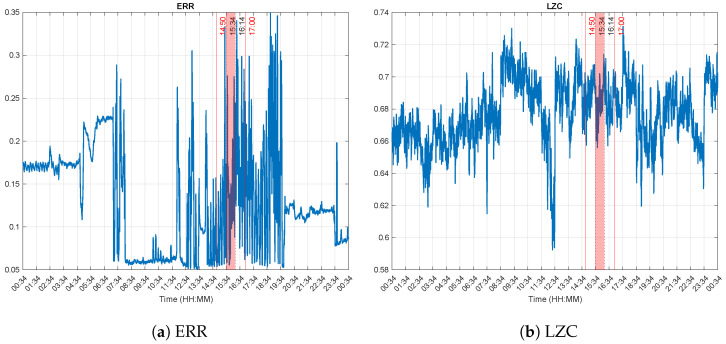
Complexity features. The experiment was performed between 14:50 and 17:00 (red vertical lines). The dotted lines at 15:34 and 16:14 delimit Δt, the time during which the experimenter reported that the patient was correctly answering the questions he was asked. In both cases, an increase was observed especially during the experiment. Higher complexity values indicate an activated cortex. These results suggest then that the patient’s consciousness level was higher at least during these times.

Figure 4 display the computed complexity features. On one hand, ERR of the Poincaré plots show a distinct decrease between 08:00 and 12:00, followed by a progressive increase, although fluctuating values, and then decrease after 19:30 (cf. Figure 4a). These values hint at an increased consciousness level, except between 08:00 and 12:00. On the other hand, the observed LZC values suggest a seemingly high signal complexity throughout the recording (Figure 4b), even more during day time. This indicates that the patient was probably consciousness throughout the 24 h.

**Figure 5:**
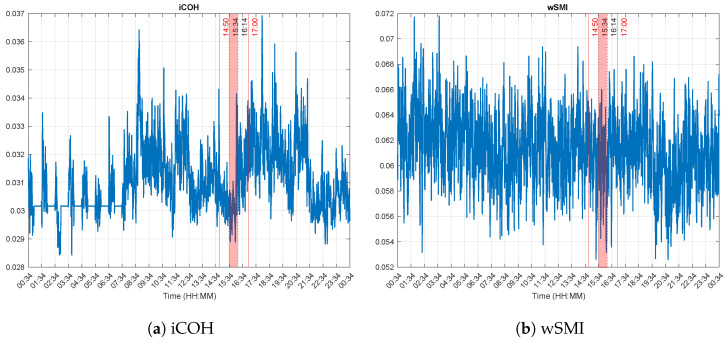
Connectivity features. The experiment was performed between 14:50 and 17:00 (red vertical lines). The dotted lines at 15:34 and 16:14 delimit Δt, the time during which the experimenter reported that the patient was correctly answering the questions he was asked. The values of the iCOH appear lower during night time (20:00 to 08:00${}^{+1}$), suggesting a lower consciousness level during that time. A decrease is also observed between 12:30 and 15:34 before slowly increasing afterwards. wSMI values on the other hand show little variations with a decreasing tendency as the time goes.

Figure 5 shows the connectivity features. The variation patterns of the iCOH during the whole recording (Figure 5a) is similar to that observed with the LZC, i.e., larger values during the day compared to the night time. It also steadily increase in the course of the experiment, and even more after, until 21:34. In the meanwhile, no much variations can be observed with wSMI as can be seen in Figure 5b.

**Figure 6:**
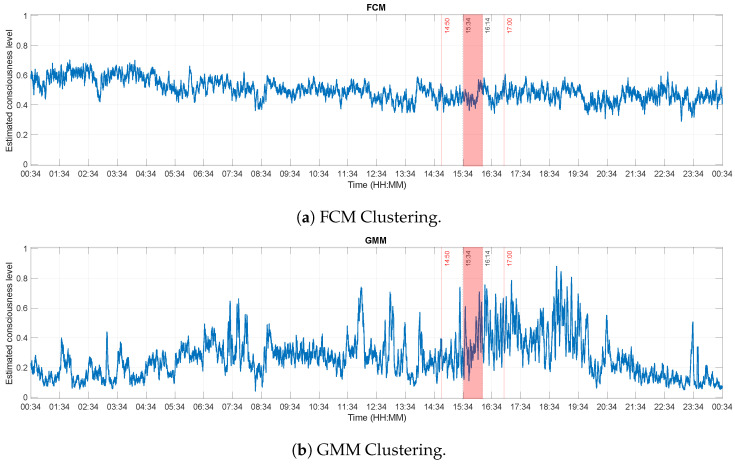
Outputs of the clustering analyses. The x-axis represents the recording times, while the y-axis displays the estimated consciousness levels. A value of 0 corresponds to unconsciousness and a value of 1 means that the patient is conscious. The experiment was performed between 14:50 and 17:00 (red vertical lines). The dotted lines at 15:34 and 16:14 delimit Δt, the time during which the experimenter reported that the patient was correctly answering the questions he was asked.

Subsequently, these features were clustered into two groups:conscious and unconscious. The size of the input vector to the soft-clustering algorithms is then 86,352 × 7 (time points × features). As mentioned earlier, the consciousness level are specified as being the degree of membership to the conscious cluster. Figure 6 shows the FCM and GMM clustering results.

FCM degrees of membership values are in average 0.4978 (cf. Figure 6a), and are comparable to the results obtained for the UWS patient in [20]. The mean value during the experiment amounts 0.4574, which is lower than the overall average. Moreover, the mean values during the night are also lower than those during the day, which suggest that the patient was more awake during night time. Times between 08:00 to 20:00 are labelled as day and 20:00 to 08:00 as night [17].

GMM degrees of membership, on the other hand, exhibit variations (cf. Figure 6b). Notably, an increase in the afternoon, and the average score during the entire recording is 0.2679. During the experiment, the mean value of the degrees of membership is 0.3594. In addition, low values are observed during the night and the highest values occur during the experiment. This may indicate a reduced consciousness during the night, but an increase one particularly at the time of experiment. Additionally, it remains high afterwards up until 19:42, with an average of **0.49**.

**Figure 7:**
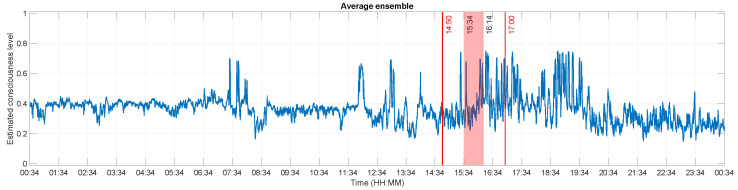
Estimated consciousness level for patient GR. The x-axis represents the recording times, while the y-axis displays the estimated consciousness levels. A value of 0 corresponds to unconsciousness and a value of 1 means that the patient is conscious. The experiment was performed between 14:50 and 17:00 (dashed lines). The dotted lines at 15:34 and 16:14 delimit Δt, the time during which the experimenter reported that the patient was correctly answering the questions he was asked. The values at these times were definitely higher than the values the rest of the time.

Figure 7 illustrates the estimated consciousness level of this patient after averaging the previous findings. Except a few surges of amplitude in the morning and at noon, the values are relatively steady and started increasing steadily as the experiment goes on and afterwards. The mean value of the estimated consciousness during the experiment and at the time he was supposed to be conscious are respectively 0.4078 and 0.4084, while the values were lower outside these time frames (cf. Table 1). These values, more particularly the increases during the experiment, imply a rise of the patient’s consciousness level. By assessing the values of the unique features extracted from the ECoG signal, it can be concluded that he was effectively conscious at least during the experiment. Indeed, out of the 18 questions asked during that time, he clearly answered 16 of them while the answers for the 2 remaining questions were unclear. This results in a 88.89% correct answer rate.

## 4. Discussion

In this paper, a method to assess CLIS patients consciousness levels using a combination of several measures is presented. By integrating multiple features, the expectation is to identify hidden characteristics that are missed by using a single feature. The objective is to maximise the probability of accurately estimating the patients’ actual state. The approach was successfully used on data from DoC patients [20]. The premise is that if it was successful in determining consciousness states in these patients, and since cognitive functions are mostly intact in CLIS patients, this approach will also be successful in determining their consciousness levels.

The results of the CLIS patient in this research showed an increased consciousness level during the experiment, in accordance to results obtained in previous researches using other features and to the observations of the experimenter. In [19], patterns suggesting a conscious state were observed in the imaginary coherence from 15:15 to 15:30 and from 16:00 to 16:10, in the multi-scale sample entropy between 15:24 and 16:14, and with Granger causality between the frontal and posterior channels from 15:34 until 16:14. Additionally, the data of this patient was analysed using a multi-scale approach associating sample entropy, permutation entropy and Poincaré plots [57]. The results reported that the patient was conscious between 16:04 and 16:10.

The different features used in this work were weighted equally and their values were normalised so to not favour any of them for the clustering analysis. Each of the features extracts a particular characteristic of the ECoG signal. The correlation between the different features and the final results (consciousness estimates) is presented in Table 2. Correlation coefficients normally range between −1 and +1. A value of 0 signifies no relationship exists between the entities [58]. Negative correlation are detected for some features. This means that although the working hypothesis stipulates that conscious states are mostly defined by increase of the features values, the latter sometimes contradict one another to some extent. For instance, $P_{theta}$ for FCM and $P_{theta}$, $P_{beta}$ and SEF95 for GMM. In the end, the algorithm appears to find a consensus between the different variations of the measures, and was able to convey the variations of the levels of consciousness that match the outcomes of the different features.

**Figure 8:**
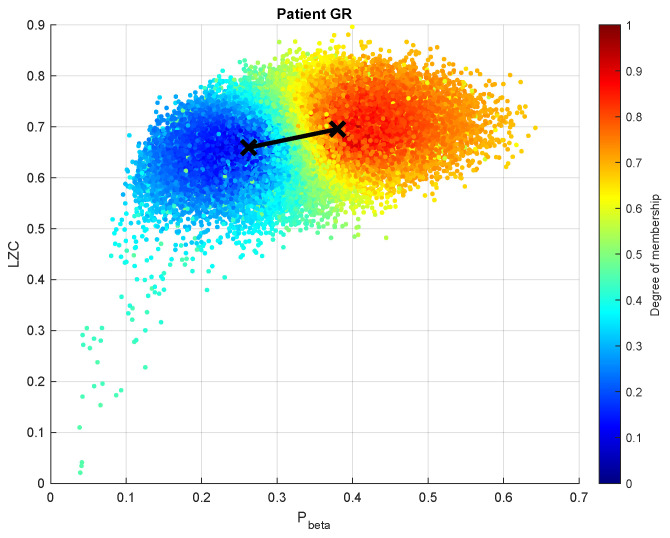
FCM clustering of $P_{beta}$ and LZC, the two features with the highest correlation coefficients to the final result. The majority of LZC values are representative of a higher level of consciousness (largely above 0.5). Ideally, data points representing unconscious states are located in the bottom left corner of the plot, while those describing conscious states are located in the top right corner.

FCM results showed a monotonous value of around 0.5 throughout the recording. This means that all the data points belong more or less equally to both clusters. In other words, the two clusters represent the same thing (cf. Figure 8 for example). This case is similar to the one encountered in [20], in which all clusters are representative of a conscious state. Consequently, the minimum and maximum possible values of the membership degrees could not be interpreted as unconscious and conscious respectively.

**Figure 9:**
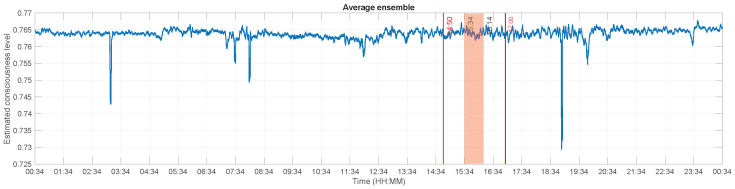
Consciousness level estimations for patient GR. The x-axis represents the recording times, while the y-axis displays the estimated consciousness levels. A value of 0 corresponds to unconsciousness and a value of 1 means that the patient is conscious. The experiment was performed between 14:50 and 17:00 (vertical dotted lines). The red shade area represent the time frame Δt during which the experimenter reported that the patient was correctly answering the questions he was asked. The average value during all the recording is 0.7638, suggesting that the patient was conscious the whole time. An increase was also observed during the experiment (mean value of 0.7640) indicating an increase of the consciousness level.

Therefore, the input vector was partitioned using pre-defined FCM cluster centres and the same Gaussian mixture model used in [20]. The obtained result is presented in Figure 9.

Table 1 summarises the average values of the estimated consciousness level during different time intervals. The values are high, with an overall mean of 0.7638. They were also slightly higher during and after the experiment. The use of predefined cluster centres determined that patient GR was conscious during the entire 24-h recording. Not only was he conscious during the whole time, but his level of consciousness also increased during the experiment. This is not implausible, since as the condition evolves, ALS-LIS patients experience an increased manifestation of insomnia [2]. An investigation of this patient sleep/wake characteristics revealed the presence of increase Slow Wave Sleep (SWS) fragmentation [17]. It is therefore highly possible that this was what happened that day.

In summary, the approach presented here was able to emulate the collective increases and decreases of the different ECoG features that indicate the patients’ consciousness levels. However, this level was not accurately estimates due to data scarcity. More precisely, this approach is proven to be working under the condition that the clusters centroids are distant from each other [20].

## 5. Conclusions

An approach to assess consciousness in a CLIS patient was presented in this paper. Different features comprising spectral, complexity and connectivity were utilised in order to increase the probability of correctly determining the patient’s actual state. Indeed, for the time being, there are no ground-truth with reference to such patients’ consciousness states. The proposed algorithm does not require active participation of the patients, and is intended to be applied on resting state data. One of the challenge faced during researches with CLIS patients is the lack of data: at least there should be enough data so that all possible states (from unconscious to conscious) are represented. An alternative to circumvent this would be to use these pre-defined clusters centroids. Research regarding consciousness of CLIS patients is also very limited. They mostly consist of the use of evoked brain potentials, P300 essentially, to distinguish them from patients with DoC; making this investigation among the first of its kind.

The presented approach can be used in addition to the traditional behavioural tests to help clinicians reduce the misdiagnosis rate of (completely) locked-in patients. Another useful application could also be to employ it as a preliminary step before initiating communication with CLIS patients. That way, it can be established only when the patient is conscious enough. Family members are primarily the ones that first discover that the patient is conscious. They are familiar with them and are consequently more likely to know how they appear when they are conscious. Future works will then use their contributions to determine the optimal threshold between conscious and unconscious states for each patient.

## Figures and Tables

**Table 1:** Average estimated consciousness level for patient GR during different time frames using (1) soft-clustering and (2) pre-defined clustering centroids.

Time	Interval	Consciousness Level (Self)	Consciousness Level (Pre-Defined)
All (24 h)	00:34 to 00:34${}^{+1}$	0.3829	0.7638
Before experiment	00:34 to 14:50	0.3879	0.7635
During experiment	14:50 to 17:00	0.4078	0.7640
“conscious” time	15:34 to 16:14	0.4084	0.7639
After experiment	17:00 to 00:34${}^{+1}$	0.3663	0.7651

**Table 2:** Spearman correlation coefficients between all features and estimated levels of consciousness. All correlation coefficients have *p*-value p≤0.05.

	FCM	GMM	Ensemble
$P_{theta}$	−0.2872	−0.403	−0.4919
$P_{beta}$	0.8906	−0.2793	0.3579
SEF95	0.3949	−0.546	0.1881
ERR	0.2812	0.165	0.3316
LZC	0.4416	0.17	0.2849
$iCOH_{theta}$	0.0203	0.0098	0.0188
wSMI	0.1428	0.346	0.3238

## Data Availability

Restrictions apply to the availability of these data. Data was obtained from Niels Bierbaumer from the Institute for Medical Psychology and Behavioural Neurobiology, University of Tübingen.

## Supplementary Materials

The entire course of the interaction between the patient and the experimenter during the experiment can be downloaded at: https://www.mdpi.com/article/10.3390/brainsci13010065/s1.

## Abbreviations

The following abbreviations are used in this manuscript:

ALS	Amyotrophic Lateral Sclerosis
BCI	Brain-Computer Interface
CLIS	Completely Locked-In Syndrome
DoC	Disorders of Consciousness
ECoG	Electrocorticography
EEG	Electroencephalography
EM	Expectation - Maximisation
ERR	Ellipsoid Radius Ratio
MCS	Minimally Conscious State
PSD	Power Spectral Density
SON	Subject’s Own Name
SSVEP	Steady-State Visual Evoked Potential
SWS	Slow Wave Sleep
UWS	Unresponsive Wakefulness Syndrome
VS	Vegetative State
wSMI	weighted Symbolic Mutual Information
