# Supplementary material for: Application of Soft-Clustering to Assess Consciousness in a CLIS Patient
Source: Brain Sci. 2022 Dec 29;13(1):65. doi: 10.3390/brainsci13010065 (PMC9856569; doi:10.3390/brainsci13010065)
Supplement: Supplementary file 1 [file brainsci-13-00065-s001.zip › brainsci-2089126-Supplementary.pdf]

Application of soft-clustering to assess consciousness in a CLIS patient.

Supplementary material: Experimental setup for patient GR

Sophie Adama, Martin Bogdan

The following reports the questions asked to the patient during the experiment, his answer, and the experimenter's notes.

The experiment started at 14:50 and ended at 17:00. + means that the patient's answer was classified as "yes", – means that it has been identified as "no".

```
Date: 2008-03-16
          Session:
          Present: Femke
           Arrive: 14.50
            Leave:

             Time:
       Heart rate: 98
Oxygen saturation: 97
    Lying on side: back/left
  Respiration BPM: 15
           Ground: S032
        Reference: G102

Initial eye movement looked much stronger than previous days. I asked
him questions (without video):
Q0.1  You feel good today? +
      You feel bad today? –

Q0.2  Are you happy that I'm here? +
      Would you prefer to be alone and to watch TV?  –

The responses were so strong that I though a conversations was
possible. I turned on the video.
The following questions were asked. Someone should check the video to
check me.

      Q1    You feel good today? +
            You feel bad today? –

      Q2    Are you German? +
            Are you Dutch? –

      Q3    Dou you feel pain? –
            Are you free of pain? +

      Q4    Are you satisfied by the health care at BS (city in
      Germany)? +
            Are you unsatisfied by the health care at BS? –

            Are you unsatisfied by the health care at BS? –
```

Are you satisfied by the health care at BS? +

Q5 Are you still happy to have decided for the operation? +
Are you sorry for having decided to do the operation? -

Q6 Do you think, you have feedback control over sound? +
Do you think the feedback is not controllable? -

Q7 Are the sounds loud enough for you? +
Should we turn the speaker louder? -
Should we turn down the speaker?
Are the speakers o.k.? +

"We would like to know about your psychological status. We want to know how your mood is."

Q8 Are you positive regarding the future? +
Do you not know exactly what future will bring for you, so, are you neutral for your future? -
Are you negative for the future? -

"I will ask you now a question, that have to be asked to you somewhen."

Q9 Do you wish sometimes, you were dead? -
You never wished to be dead? +##

Q10 Can you enjoy your life under these circumstances? +
You don't enjoy your life any more? -

Q11 Was it a good decision to bring you to BS? +
Do you prefer to go back to Vohenstrauss (his home town)? +&- (not clear)
Do you prefer to go back to Vohenstrauss? -

Q12 Do you want to stay at BS in the future? -
Do you want to stay at BS in the future? -
Do you prefer to go into another nursing home? +
Do you prefer to go into another nursing home? +

Q13 You want to go back to Vohenstrauss in some later period? Do I understand you correctly? + (no clear answer)
You want in another nursing home than Vohenstrauss? + (no clear answer)

" I have been informed, that our political contacts we have engaged to convince the health insurance regarding your care costs will publish a report about you in the SPIEGEL (very renown German journal). Unfortunately, I don't know more than that, neither about the content of the article. I will inform myself to tell you."

Q14 Shouldn't we go to public with the heath insurance? -
Shouldn't we fight we the health insurance? -
Should we fight with the health insurance? -

Q15 For long term schedule: You want to go to Vohenstrauss? +
For long term schedule: You want to stay at BS? +

"It's not clear to me what you want to say, GR."

Q16 You don't care where you will be in future? -

“I ask you a different way now.”

Q17 For long term schedule, are you Prefer Vohenstrauss before BS? –
“I see a NO in your eyes, is this correct?”
\+ (including corner of the mouth)

(I tried letting him repeat the mouth twitch but it didn"t always work. Instructed hi to use whatever he could
or both).

Q17 Do you prefer, for long term, to stay at BS before Vohenstrauss? –
You want another nursing home than those 2?

You want to spell something?

George then spelled the word "AMBERG"
This was a long process where I repeatedly double checked (verified "correct?" and "wrong?") if I got all letters correct. I also asked him for confirmation or declination of the final word "AMBERG". Someone should CHECK the video and check me.
I, then suspected that he wanted to go to Amberg instead of stazying in BS or going back to Vehenstrauss.

Q18 You mean, you want to go to Amberg in the future? +
Did I understood you wrong, that you want to go to Amberg in the future? –

VIDEO lost power. We take a break and I type up this conversation.

Time: 17.00
Heart rate: 94
Oxygen saturation: 98
Lying on side: back/left
Respiration BPM: 15
Ground: ?
Reference: ?
